# Supplementary material for: Solar energy and regional coordination as a feasible alternative to large hydropower in Southeast Asia
Source: Nat Commun. 2021 Jul 6;12:4159. doi: 10.1038/s41467-021-24437-6 (PMC8260807; doi:10.1038/s41467-021-24437-6)
Supplement: Supplementary file 1 — Supplementary Information [file 41467_2021_24437_MOESM1_ESM.pdf]

Supplementary Information for: Solar energy and regional coordination as a feasible alternative to large hydropower in Southeast Asia

Kais Siala
TUM CREATE Ltd., Singapore

AFM Kamal Chowdhury
Environmental Studies Department,
University of California Santa Barbara, Santa Barbara, California
Pillar of Engineering Systems and Design
Singapore University of Technology and Design, Singapore

Thanh Duc Dang
Pillar of Engineering Systems and Design
Singapore University of Technology and Design, Singapore

Stefano Galelli
Pillar of Engineering Systems and Design
Singapore University of Technology and Design, Singapore
Email: stefano_galelli@sutd.edu.sg

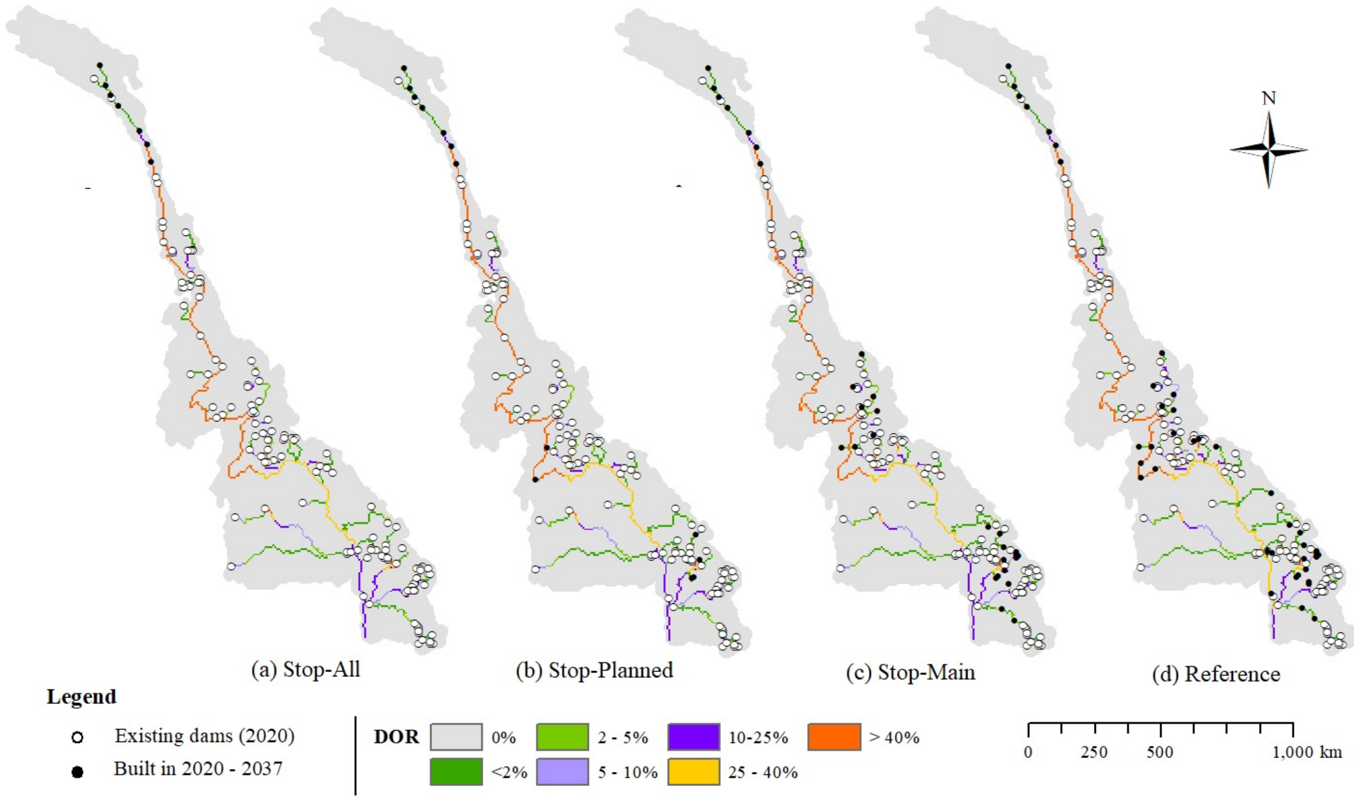

Figure S1: Effect of dams on river flow. Change in the Degree of Regulation (DOR) between the four dam development portfolios considered in this study—Stop-All (a), Stop-Planned (b), Stop-Main (c), and Reference (d). All values are calculated for the year 2037. Existing dams (as of 2020) are represented by white circles.

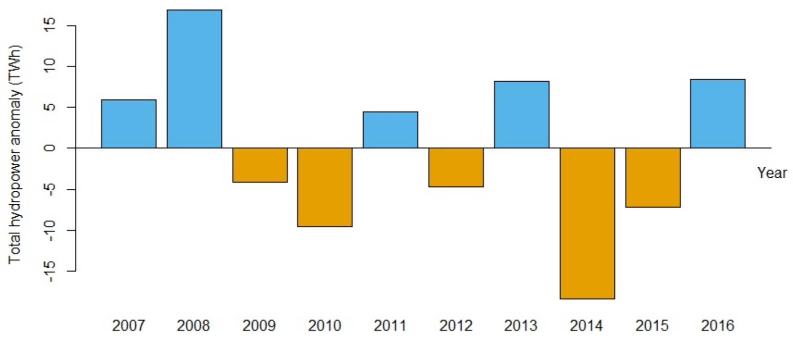

Figure S2: Annual anomalies of hydropower production (in TWh) for all dams in the Lower Mekong region over the period 2007–2016. The data are generated via simulation with VIC-Res, using the hydropower fleet operational in 2016. The anomalies are calculated with respect to the average annual hydropower production, which is equal to 142.2 TWh.

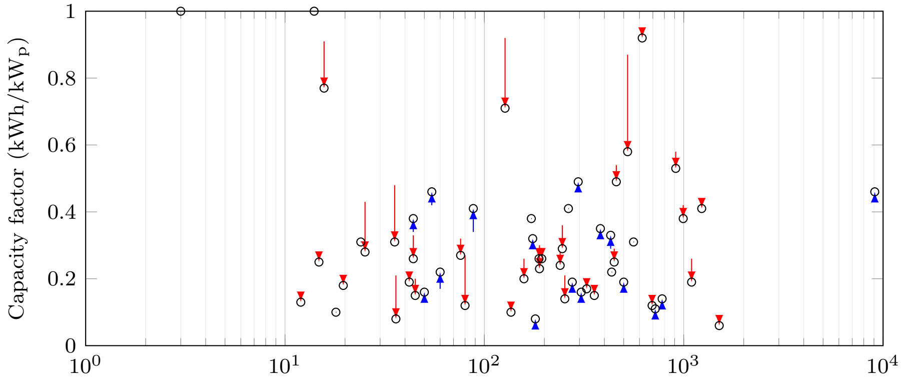

(a) Change in capacity factors for a dry year (2014)

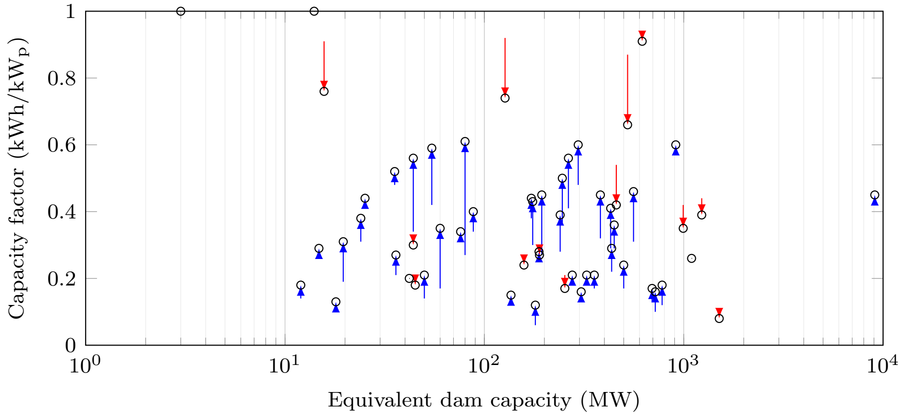

(b) Change in capacity factors for a wet year (2008)

Figure S3: Effect of hydro-climatic variability on the capacity factor of all dams. Each black circle represents the capacity factor of one dam during the "dry" (2014) and "wet" (2008) years (upper and lower panels, respectively). The horizontal coordinate of each point corresponds to the equivalent dam capacity (further details on the difference between existing and equivalent capacity are reported in the next figure). As expected, the capacity factor of most dams decreases in the dry year. This is visible in the upper panel, where the red arrows pointing downwards illustrate the decrease of capacity factor w.r.t. the representative, or average, year (2015). The opposite situation is depicted for the wet year, when most of the dams exhibit an increase in their capacity factor. In either case, there are dams with counter-intuitive behavior. This phenomenon is explained by the fact that the dry, average, and wet year designations describe a general pattern for the whole region—so there can be isolated areas experiencing different hydro-climatological conditions.

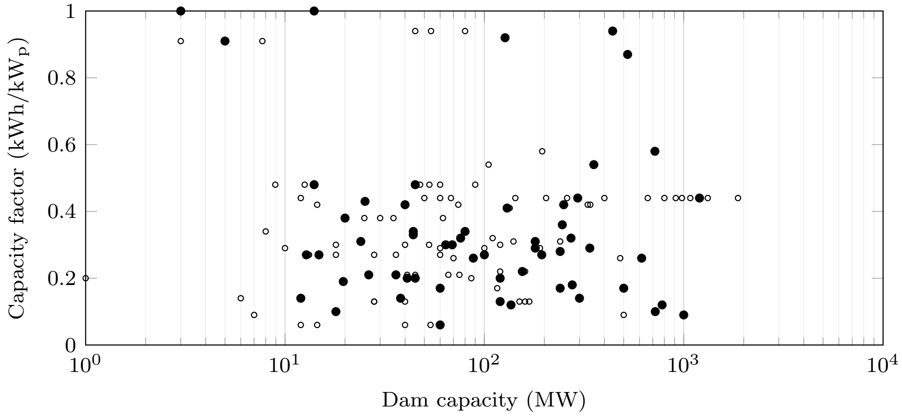

(a) Dams modeled in VIC-Res (black filled dots) and remaining dams (black circles)

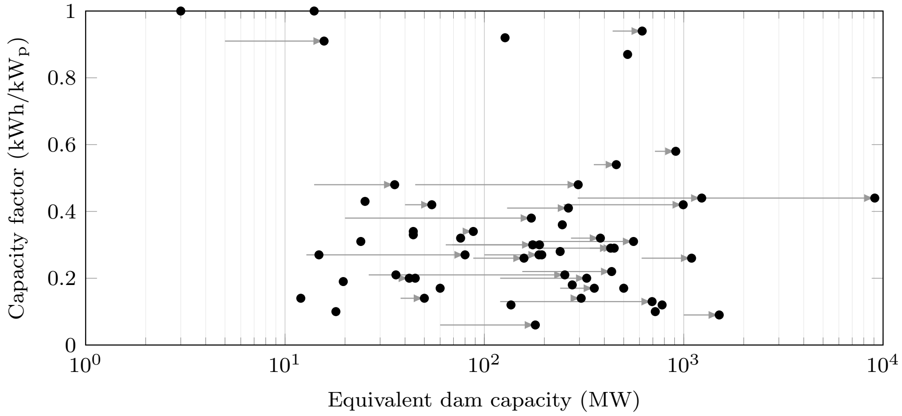

(b) Equivalent dams after combining power plants with the same time series

Figure S4: The capacity expansion model requires hydropower profiles for all dams built or planned over the period 2016–2037 (in Thailand, Laos, and Cambodia). The dams built and operated by the year 2019 are explicitly modelled in VIC-Res. Their capacity factor (for the 2015 hydro-climatological conditions) is represented in the upper panel by the black filled dots. For the other dams (under construction or at different planning stages), it is not possible to run a simulation with VIC-Res, because there are not detailed design specifications available. We therefore used a proximity search to identify for each planned dam the most similar existing dam (in terms of location and installed capacity), from which the planned dam inherits the hydropower profile. The so-determined capacity factors of these planned dams are represented by the black circles. Note that the horizontal coordinate of each point corresponds to the installed capacity of a dam, either existing or planned. In the bottom panel, we report the capacity factor of the equivalent dams, whose capacity (horizontal axis) is obtained by summing the capacity of an existing dam to the capacity of one, or multiple, planned dams allocated to it through the proximity search. The change from existing to equivalent capacity is illustrated by the grey arrows.

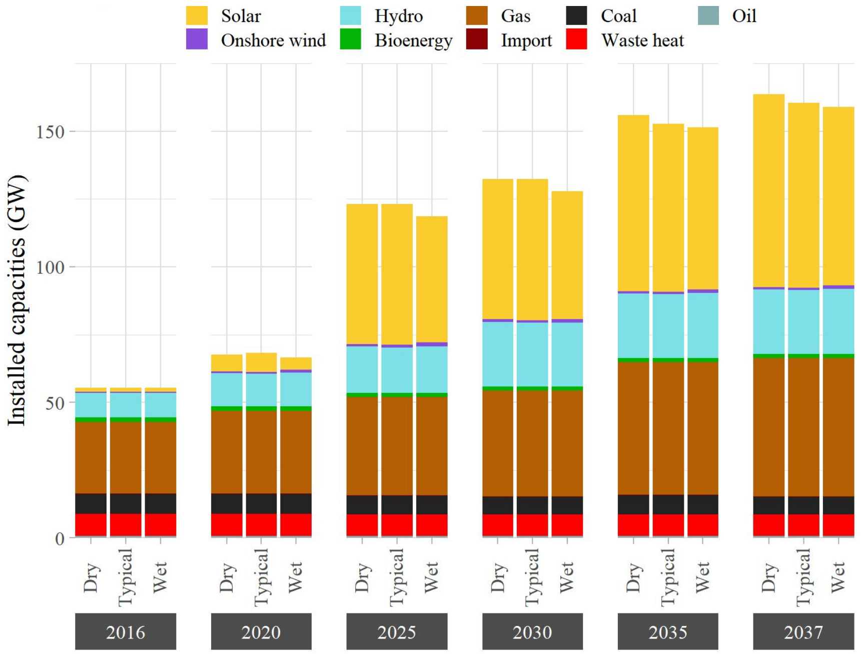

Figure S5: Sensitivity of the capacity expansion plans to hydro-climatological conditions. The capacity expansion plans are informed by representative hydropower profiles simulated by VIC-Res for an average year (2015). Since hydropower production exhibits inter-annual variability, one may expect that the plans may be sensitive to varying hydro-climatological conditions. To test this hypothesis, we run urbs under dry (2014) and wet (2008) conditions, and compare the evolution of the installed capacity against the one attained with average conditions. Despite the differences in hydropower profiles, the installed hydropower capacity shows limited changes between the scenarios (Dry: 23.8 GW, Wet: 24 GW). Note that the decrease of installed hydropower capacity in the dry scenario is offset by an increase of about 5 GW of installed solar PV. Overall, we conclude that the results related to the cost-optimal hydropower capacity are robust w.r.t. the hydro-climatic variability affecting the region.

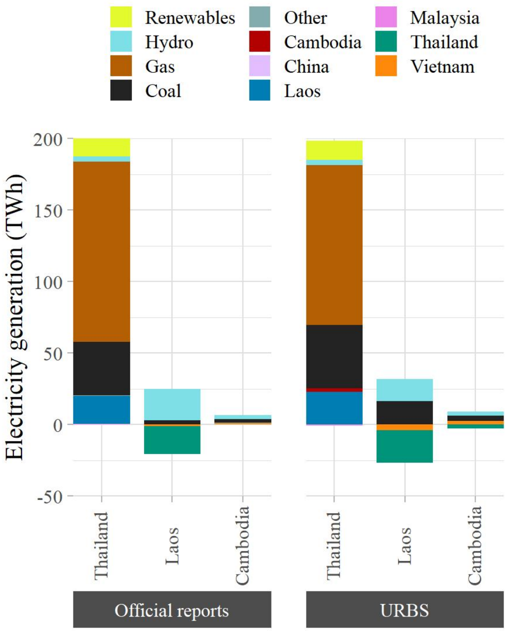

Figure S6: Validation of the electricity generation mix for the year 2016. A capacity expansion exercise must build on a correct representation of the existing power supply dynamics. Here, we show that the setup of urbs for the year 2016 accurately reproduces the electricity generation mix reported by the energy authorities [1, 2, 3, 4]. The contribution of coal in the power mix is slightly overestimated because the optimization model has a perfect foresight and does not reflect unexpected events like outages, grid congestion, and fuel cost fluctuations, which usually require the use of gas and/or oil-fired power plants because they can ramp up/down their capacities faster.

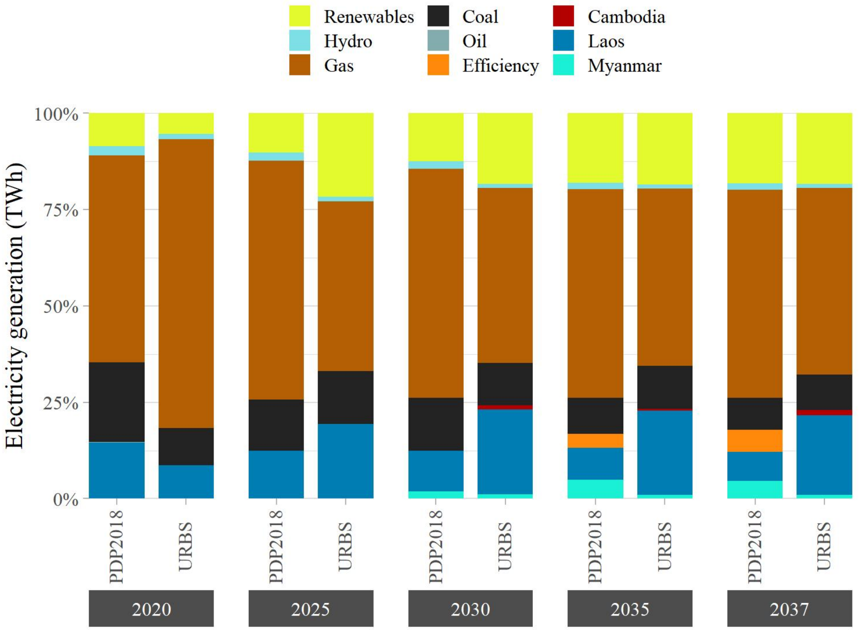

Figure S7: Validation of the capacity expansion plans. A comparison between the Thai electricity generation mix projected by urbs and the Power Development Plan (PDP2018) shows that the model diverges in the early time steps by underestimating the renewable expansion and relying on more gas for decarbonization, then flips in 2025 and overestimates the contribution of non-hydro renewable energy, to finally settle on the same level after 2035. The PDP2018 projects a more diversified mix by 2035, by importing more hydro from Myanmar and investing in efficiency improvements, which is not modeled in urbs. The latter projects ever increasing imports from Laos. Overall, the model manages to match the share of non-hydro renewable energy, but probably overestimates the imports from Laos (which are mostly hydropower).

Table S1: Cost assumptions for key technologies.

**Technology**	**Year**	**Inv. costs** [US$/MW]	**Fixed costs** [US$/MW]	**Var. costs** [US$/MWh]
Gas combined cycle	all	850000	21250	2
Hydro	all	1793000	30302	0
Solar PV	2020	800000	13600	0
	2025	720000	12240	0
	2030	640000	10880	0
	2035	610000	10370	0
	2037	595000	10115	0
Wind onshore	2020	1350000	36450	0
	2025	1325000	35775	0
	2030	1300000	35100	0
	2035	1250000	33750	0
	2037	1230000	33210	0

Table S2: Input data for VIC and VIC-Res models.

**Category**	**Dataset**	**Ref.**
DEM	Global 30 Arc-Second Elevation (GTOPO30)	[5]
Land use	Global Land Cover Characterization	[6]
Soil	Harmonized World Soil Database	[7]
Precipitation	Global Meteorological Forcing Dataset	[8]
Temperature	Global Meteorological Forcing Dataset	[8]
Reservoir surface extent	Landsat TM and ETM+	
Hydropower dam	Mekong River Commission (MRC) dam database, Electricity Generating Authority of Thailand (EGAT) database, Global Reservoir and Dam Database, International Commission on Large Dam's database, and Water, Land, and Ecosystem (WLE)'s database	[9, 10, 11, 12]

Table S3: Major inputs and constraints for the power system model in urbs.

**Category**	**Dataset/Values**	**Ref.**
Commodity costs	Bioenergy: Levelised Costs of Electricity for Renewable Energy Technologies in ASEAN Member States II; Coal, Gas, Oil: approximate cost from the World Energy Outlook 2019; Waste heat: own assumption	[13, 14]
Technology costs	Battery storage, transmission lines, and all power plants except hydropower: directly retrieved or interpolated from the Energy Technology Reference Indicator projections for 2010-2050; Hydropower dams: Levelised Costs of Electricity for Renewable Energy Technologies in ASEAN Member States II	[15, 13]
Emission factors	Values in tCO_2/MWh – Coal: 1.04, Gas:0.47, Oil: 0.73, Other: 0.0	[16]
Installed capacities and demand profiles	2016's annual reports of EDC, EDL, and EGAT; Energy statistics of Thailand 2017	[1, 2, 3, 4]
Planned expansion and demand growth	Thailand Power Development Plan 2018–2037	[17]
Wind and solar time series	python Generator of REnewable Time series and mAps (pyGRETA) using weather reanalysis data from MERRA-2 and other datasets	[18]
CO_2 constraints	Power Development Plan 2018-2037 (extrapolated the CO_2 intensity of Thailand to the whole region)	[17]

References

[1] EDC. Annual report 2016. Technical report, Electricite Du Cambodge (EDC), Phnom Penh, Cambodia, 2016.

[2] EDL. Annual report 2016. Technical report, Electricite Du Laos (EDL), Vientiane Capital, Lao PDR, 2016.

[3] EGAT. Annual report 2016. Technical report, Electricity Generating Authority of Thailand (EGAT), Nonthaburi, Thailand, 2016.

[4] EPPO. Energy statistics of Thailand 2017. Technical report, Energy Policy and Planning Office (EPPO), Ministry of Energy, Bangkok, Thailand, 2017.

[5] U.S. Geological Survey's Center for Earth Resources Observation and Science (EROS). Global 30 arcsecond Elevation, 1996. Accessed 04/06/2018.

[6] U.S. Geological Survey (USGS). Global Land Cover Characterization Dataset, 1992. Accessed 03/06/2018.

[7] Guenther Fischer, Freddy Nachtergaele, Sylvia Prieler, HT Van Velthuizen, Luc Verelst, and David Wiberg. Global agro-ecological zones assessment for agriculture (gaez 2008). IIASA, Laxenburg, Austria and FAO, Rome, Italy, 10, 2008.

[8] Justin Sheffield, Gopi Goteti, and Eric F Wood. Development of a 50-year high-resolution global dataset of meteorological forcings for land surface modeling. Journal of climate, 19(13):3088–3111, 2006.

[9] Mekong River Commission (MRC). MRC hydropower database, 2017. Accessed 17/03/2021.

[10] EGAT. Exploring EGAT dams and thermopower plants. Technical report, Electricity Generating Authority of Thailand (EGAT), 2018. Accessed 17/08/2021.

[11] Bernhard Lehner, Catherine Reidy Liermann, Carmen Revenga, Charles Vörösmarty, Balazs Fekete, Philippe Crouzet, Petra Döll, Marcel Endejan, Karen Frenken, Jun Magome, et al. High-resolution mapping of the world's reservoirs and dams for sustainable river-flow management. Frontiers in Ecology and the Environment, 9(9):494–502, 2011.

[12] Water, Land and Ecosystems (WLE) in the Greater Mekong. WLE Hydropower database, 2020. Accessed 09/08/2020.

[13] The ASEAN-German Energy Programme (AGEP) Team. Levelised Costs of Electricity for Renewable Energy Technologies in ASEAN Member States II. Technical report, ASEAN Centre for Energy (ACE), Jakarta, Indonesia, 2019. Accessed 2020/12/22.

[14] International Energy Agency. World Energy Outlook 2019. 2019.

[15] Roberto Lacal Arantegui, Arnulf Jaeger-Waldau, Marika Vellei, Bergur Sigfusson, Davide Magagna, Mindaugas Jakubcionis, Maria del Mar Perez Fortes, Stavros Lazarou, Jacopo Giuntoli, Eveline Weidner Ronnefeld, Giancarlo De Marco, Amanda Spisto, and Carmen Gutierrez Moles. ETRI 2014 - Energy Technology Reference Indicator projections for 2010-2050. EUR - Scientific and Technical Research Reports, Joint Research Center of the European Union, 2014.

[16] Moti L Mittal, Chhemendra Sharma, and Richa Singh. Decadal emission estimates of carbon dioxide, sulfur dioxide, and nitric oxide emissions from coal burning in electric power generation plants in India. Environmental Monitoring and Assessment, 186(10):6857–6866, 2014.

[17] EPPO. Thailand power development plan 2018-2037 (PDP2018). Technical report, Energy Policy and Planning Office (EPPO), Ministry of Energy, Thailand, 2018.

[18] Kais Siala and Houssame Houmy. tum-ens/pyGRETA: python Generator of REnewable Time series and mAps, June 2020.
